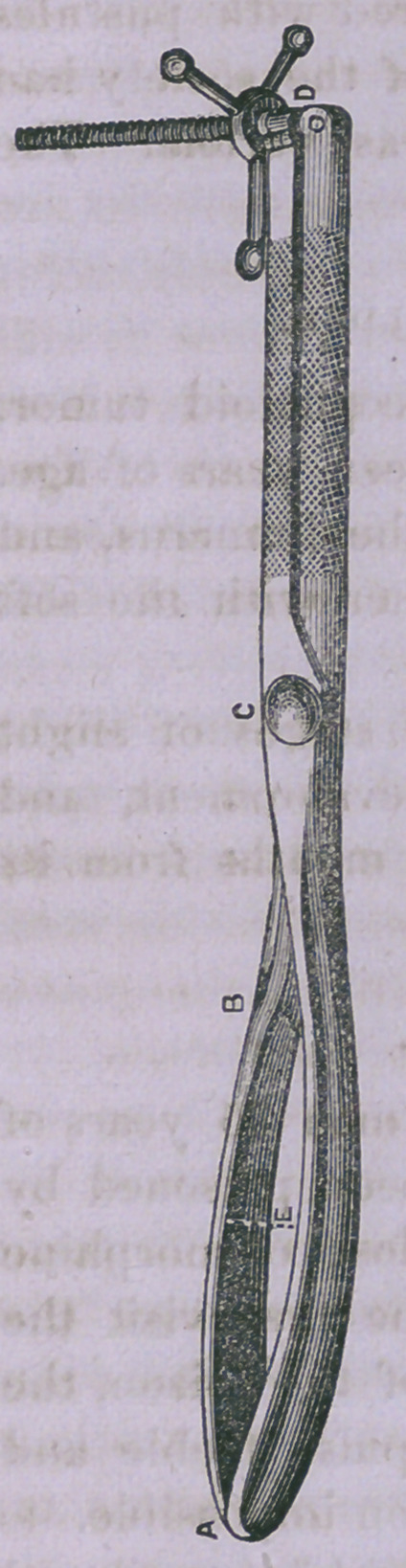# Proceedings of the Chicago Medical Society

**Published:** 1868-05-15

**Authors:** 


### PROCEEDINGS OF THE CHICAGO MEDICAL SOCIETY.

THE ANNUAL MEETING of the Society was held on the evening of April 2nd. The following members were elected officers for the ensuing year:

E. Marguerat, M.D., *President.*

R. G. Bogue, M.D., *Vice President.*

P. S. McDonald, *Secretary and Treasurer.*

*Committee on Ethics*—Drs. N. S. Davis, G. C. Paoli, J. P. Ross.

*Censors*—Drs. S. Wickersham, J. Reid, N. Loverin.

*Committee on Sanitary Condition of City*—Drs. I. Hatch, D. B. Trimble, T. P. Seeley.

### Singular Case of Exophthalmos.

Dr. Holmes presented a male patient, 33 years of age, affected with a rare form of exophthalmos of the right eye, which supervened fifteen years ago upón a punctured wound of the left eye. This eye inflamed extensively, and in a few weeks became atrophied.

The right eye became inflamed and somewhat prominent. The exophthalmos soon presented the appearance observed in the eye at the presént time. The protrusion seems to depend upon a partial paralysis of the recti and orbicularis muscles. It is so extensive that the patient can not close the lids; on pressing two fingers of each hand into the upper and lower portion of the orbit, the eye is easily, and without pain, forced almost completely out of orbit. Quite gentle pressure upon the lids causes the globe to recede to its normal position. There is no symptom of pulsation or of a tumor in the orbit. The patient's health has always been remarkably good, with no evidence of disease of the heart, or of the thyroid gland.

Long exposure of the anterior portion of the globe to the air has produced thickening of the conjunctiva, and also opacity of the cornea, the upper fourth of the latter, however, being simply nebulous.

There is scarcely any secretion from the lachrymal gland and conjunctiva. The motions of the globe are very limited, being more extensive upward and downward, than in any other direction. The oblique muscles seem to have almost wholly lost their power, since the rotation of the globe on its antero-posterior axis is scarcely perceptible.

An elastic suspensory bandage around the head, so placed on the lower lid as to support the globe, not only partially covered the cornea, but also greatly reduced the irritation.

The patient has been practically blind for fifteen years. There is evidently some lesion of the optic nerve, or retina, since he could otherwise see the outlines of large figures through the upper border of the cornea more distinctly than he now does.

### Polypoid Tumor of the Cornea.

Dr. Holmes also presented a large polypoid tumor, which he had removed from the centre of the cornea of a young woman, who had suffered two years from "granulated lids," and pannus of the right eye. The tumor was somewhat flattened by the pressure of the lids, and very movable, being attached to the cornea by a narrow peduncle. It was readily excised by means of a small pair of scissors, after it had been slightly elevated from the cornea by a pair of forceps. Two weeks after the operation, the eye bore well the usual astringent applications, which slowly improved the condition of the cornea.

The microscopic appearances of the tumor, as described by Drs. Hunt and Lyman, are similar to those of fibrous polypus, the fibrous elements being somewhat indistinct, but enclosing a large number of cells, resembling epithelial cells. In some portions of the tissue were numerous collections of fine granular substance, containing large, irregular, nucleated cells.

### Large Polypus Nasi.

Dr. Bogue exhibited a remarkably large polypus, which he had removed from the nose of a boy twelve years of age. The tumor extended so far into the throat, that the act of coughing would force the pendulous portion forward nearly to the teeth. The attachment was very narrow, rendering the removal of the whole tumor exceedingly easy.

### Cephalotribe.

Dr. Paoli presented a form of this instrument, which he had devised, differing from that of Prof. Hodges in the following particulars: It is nearly a pound lighter; its blades, five inches shorter, their tips coming in contact with each other instead of being separated. The instrument is sufficiently strong to press out the contents of the head, and to retain so firm hold as to enable the obstetrician to draw down the fœtus without the necessity of changing instruments.

[The following is Dr. Paoli's description and cut of the instrument.—ED.]

It is an acknowledged fact, that of late years, on the continent of Europe, the Cephalotribe has been preferred to the old instruments, in performing embryotomy.

Many of the profession have had prejudices against the above instrument, on the grounds, that it was so heavy and clumsy, and that its use was attended with great difficulties.

In improvements on this valuable instrument, our distinguished American accoucheur, Professor Hodge, stands foremost. As an improvement on Professor Hodge's, I have constructed one (in consultation with our excellent German instrument maker, Degenhardt, here, in Chicago) which, as will be easily seen by the description, is not only lighter, and easier in its application, but also rotations of the fœtus' head can be performed in the smallest space of the pelvis.

The instrument being made of strong steel, well tempered, is capable, under the influence of the screw, to compress any fœtal head.

The whole weight of the instrument is two pounds and a half.

From A to B seven inches; from A to C, 11 inches; from

C to D, 8 inches. The width of the blade, one inch. Horizontal curve, one inch. The space between the blades of the widest part, when the instrument is closed, is only one inch.

### Small Pox under Rare Circumstances.

Dr. Wickersham stated that he had lately successfully vaccinated a mulatto girl, aged nineteen, unmarried, but pregnant, who had just been exposed to small pox. She had never been vaccinated before. Four weeks after the vaccination, a well developed child was born, covered with pustules of about the eighth day. Other members of the society had examined the child, and pronounced the disease variola. The case terminated favorably.

### Encephaloid Disease of Shoulder.

Dr. Seeley exhibited a portion of an encephaloid tumor, from the shoulder joint of a girl only nineteen years of age. The tumor involved the upper portion of the humerus, and the articular portion of the scapula, together with the soft portions of the arm and neck.

The growth passed through the various stages of slight swelling, ulceration, extensive fungus development, and caused the death of the patient in about two months from its first appearance.

### Poisoning from Morphine.

Dr. Merriman related a case in which a man 35 years of age, suffering from delirium tremens, had been poisoned by taking very much larger and more frequent doses of morphine than his physician had prescribed. At the first visit the patient was found fully under the influence of the poison, the breathing being very slow and stertorous, pulse feeble and frequent, lips livid, skin moist, and deglutition impossible.

Electricity was found to be the only agent which produced any decided effect upon the patient. While this caused a very marked increase in the number of the respirations, and force of the pulse, for a short period, the patient finally succumbed in about five hours after the first visit.

### PROGRESSIVE PARALYSIS OE THE INSANE.

(PARALYSIE GÉNÉRALE DES ALIÉNÉS OF THE FRENCH.)

BY A. W. BOSWORTH, M.A., PARIS, FRANCE.

Thesis for the Degree of M.D., Presented to the Faculty of the Rush Medical College, Chicago.

(*Continued from page* 299.)

In other cases the intellectual faculties appear to have been the more restored, while the impediment of speech very apparently remains. Then the patient, although he has again taken his accustomed place in society, plays his part in the worldly routine of life, for the time being, in a consistent manner; now asserts that all is well, yet, to the physician must he present a grave situation. The latter must be ready to counsel him and his friends; must be prepared for any medico-legal investigations, and rest assured that, if he has truly diagnosed the patient to have presented the characteristic symptoms of general palsy, the medical science of to-day requires him to know, that sooner or later there will be a relapse, and death will be the final, inevitable termination of the patient.

These remissions are of various length. Calmeil declares to have seen the malady thus remain absolutely stationary ten, fifteen, or twenty-four months. M. Marcé says that he has seen them prolonged eighteen months; one case more than two years, another more than five.

M. Marcé, to whom we are indebted for many of the ideas presented in this thesis, in his publication of "Mental Diseases," speaking of an abnormal termination of general palsy, says, "We see patients having presented ambitious delirium, impediment of speech, and all the symptoms of general palsy, remain in a state of dementia, while still preserving isolated, ambitious ideas, but offering a complete arrestation in the development of the troubles of motility. Life then may be indefinitely prolonged, as in chronic mania, or in simple dementia. I have at this moment (1862) two patients who present, one since six years, the other since eight years, this singular transformation. Let us say concerning the subject, to explain my belief in regard to it, that these two patients had to the greatest degree, taken alcoholic drink to an excess, and that I am disposed to regard the facts of this nature, not as veritable general palsy, but as cases of alcoholic dementia, associated with ambitious ideas and with transient troubles of motility only due to a special intoxication."

Another reason for the great variety of the duration of this disease is what has been considered by some authors as a complication of the malady; by others as a predisposing cause; by others as its first symptom; we mean, congestion.

All physicians admit that there are seen in the precursory period, or especially in the two last periods, congestions more or less intense. Many authors, with Bayle, consider the cerebral congestions as constant in general palsy. Their character is to be sudden. Most usually they are preceded or accompanied by change of character if occurring in the precursory period; but sometimes the malady begins with a violent congestion of an apoplectic or convulsive form; or such an attack has preceded the disease a few weeks or months; or such congestion may rapidly cause death at an advanced period.

Such being the case, it is necessary before giving the prognosis to be informed in regard to the character the congestion assumes with each patient.

Some authors give three degrees, or different forms of congestion; others eight; some six. With the latter we have found the following characteristics in the book of M. Marcé, who thus enumerates them: "first degree, slight, with excitation; second degree, maniacal; third degree, comatose; fourth degree, hemiplegic; fifth degree, convulsive, apoplectiform; sixth degree, irregular form." In the first form there is a simple increase of the circulation, evident from acceleration of the pulse, redness of the face, intellectual and physical excitement; augmentation in the impediment of speech, and trouble of motility, if these latter are already manifest. This attack may last from a few hours to two or three days.

The second form differs from the first only by being more intense, and causing the patient to be violent and maniacal.

In the third form, somnolence, redness of the face, physical and intellectual weakness; the comatose state advancing to insensibility, finally terminating by aggravation or amelioration of the symptoms.

The fourth form, suddenly appearing, may throw the patient into some ordinary state of hemplegia for a few hours or days, and usually disappears mostly, but leaves the patient in an aggravated condition.

The fifth is really apoplectiform, or convulsive, and is the most dangerous; often the most frequent, immediately throwing its victim into a state insensible to all excitants, in which condition he may immediately expire; or later, be seized with general or partial convulsions, as in epilepsy. The attacks of this form may be quite numerous in the course of twenty-four hours, being separated by more or less complete coma. In some cases the muscular fibres are, as it were, continually contracted in a spasmodic manner for hours. These attacks, if occurring at five or six different periods of the year, soon produce death. If not speedily mortal, they disorganize more or less the nervous elements, leaving more or less accentuated contractions of various parts, and perceptibly diminishing the sensibility. These attacks often so closely resemble those of epilepsy, that naught but the antecedents of the patients, the duration of the coma, being prolonged sometimes for hours or days, and the repeated succession of the attacks, give them their true nature.

It has been observed that some patients can support thirty or forty of these epileptiform attacks without appearing to be greatly enfeebled, when suddenly another may come, and leave them a corpse.

The sixth form offers a mixture of all the preceding, which may alternate with the same patient. As congestions are so liable to occur during the march of general palsy, the attention of the physician should ever be on the alert, endeavoring to remove all that may tend to favor their apparition; and he should remember that although they usually present the symptoms of a greatly increased circulation, yet they may assume a syncopal form.

### Causes.

They have been divided into predisposing and occasional. This is the division that has been adopted by all authors who have treated of mental diseases. It is probable that all the causes given for madness may be applied to general palsy, but I shall especially insist on those which have an intimate relation with this disease.

Mr. Parchappe divides them into moral and psychical.

Mr. Baillarger classes among the predisposing causes, hereditability, temperament, age, professions, climate, and seasons.

Among the occasional, cerebral congestions, sanguinary suppressions, excesses in drinking. We shall follow this division, and also add that of onanism, venereal excess, and refer to syphilis and pellagra, which are given by some authors.

### Predisposing Causes.

*Hereditability.*—Inheritance of disease is only a particular form of general heredity, a too frequent result of that invariable law which causes anatomical elements to have the property of directly giving birth to like elements.

At the first thought it would seem that statistics should be the only positive basis to mathematically establish the greater or less frequence of disease, and especially of the cause we are now treating; yet if we glance over the statistical tables of various authors, we are perplexed by finding that results are as various as opinions. These differences, however, are readily explained by the variable bases that have been adopted to establish the statistics.

(*To be continued.*)

## Figures and Tables

**Figure f1:**